# Supplementary material for: Occurrence of Ocular Disorders in California Sea Lions Under Human Care: Comparing Freshwater and Saltwater Housing Conditions
Source: Animals (Basel). 2025 Mar 5;15(5):739. doi: 10.3390/ani15050739 (PMC11899396; doi:10.3390/ani15050739)
Supplement: Supplementary file 1 [file animals-15-00739-s001.zip › animals-3431212-supplementary.pdf]

Supplementary Material

Brehm et al. 2025, Occurrence of Ocular Disorders in California Sea Lions Under Human Care: Freshwater Versus Saltwater Housing, Animals MDPI

Results including animal B05798 (female, saltwater housing). This animal had chronic eye disorders:

We calculated the occurrence of specific diagnoses (expressed as a percentage per month over a ten-year period) based on the time the animals spent in each facility. For each month of the year, we derived average occurrences of various diagnostic categories, averaged over ten years (Figure A1). We compared these occurrences across all months and diagnostic categories and found no significant difference between the saltwater and freshwater facilities for eye-related diseases (Wilcoxon Signed-Rank test; freshwater (Mdn = 1.5, n = 12), saltwater (Mdn = 3.4, n = 12), W+ = 44, $p = 0.724$, $r = 0.11$). Similarly, there was no significant difference in miscellaneous diagnoses between the freshwater (Mdn = 3, n = 12) and saltwater (Mdn = 2.7, n = 12) facilities (W+ = 21, $p = 0.170$, $r = 0.408$). However, we did find a significant difference in injuries between the freshwater (Mdn = 1.5, n = 12) and saltwater (Mdn = 3.4, n = 12) facilities (W+ = 51, $p = 0.019$, $r = 0.71$). The skin disease category had too few samples for a Wilcoxon Signed-Rank test.

To further explore whether the occurrence of different diseases varied by month, we conducted a chi-square test of independence. Figure A2A indicates that skin diseases are most frequently diagnosed in January, eye diseases peak in July, and injuries occur often in February. However, the residuals from expected values were comparatively low (maximum = 2.45), therefore there was no significant association between diagnostic category and month for the freshwater facility ($X^2 = 33.097$, df = 33, $p = 0.4625$). Figure A2B reveals that skin diseases are predominantly diagnosed in January (similar to the freshwater facility), while eye diseases show small peaks in November and May. Because the residuals in this case were comparatively high (maximum = 5), we found a significant association between diagnostic categories and month for the saltwater facility ($X^2 = 72.111$, df = 33, $p < 0.0001$). When cumulative occurrences of diagnostic categories were directly compared between the freshwater and saltwater facility (see Figure A2C), it became evident that injuries are overrepresented in the saltwater facility, while misc. diagnoses are are above the expected value in the freshwater facility. Despite this observation, the residuals were also relatively small (maximum = 1.5), resulting in no significant association ($X^2 = 7.0902$, df = 3, $p = 0.06908$). The residuals for eye diseases are around zero for the fresh and the saltwater facility.

We also tested if the ocular diseases were occurring more frequently during times where sunlight exposure (hours) were higher (Fig. A3). Interestingly we found a significant difference in the frequency of ocular diseases between fresh and saltwater (Wilcoxon Signed-Rank test; W+ = 115.5, $p = 0.01332$, $r = 0.384$). Ocular diseases were diagnosed more often during periods with higher sunlight exposure (hours) for the freshwater facility (Mdn = 256.5), whereas ocular diseases were diagnosed more often during periods with less light for the saltwater facility (Mdn = 145.8).

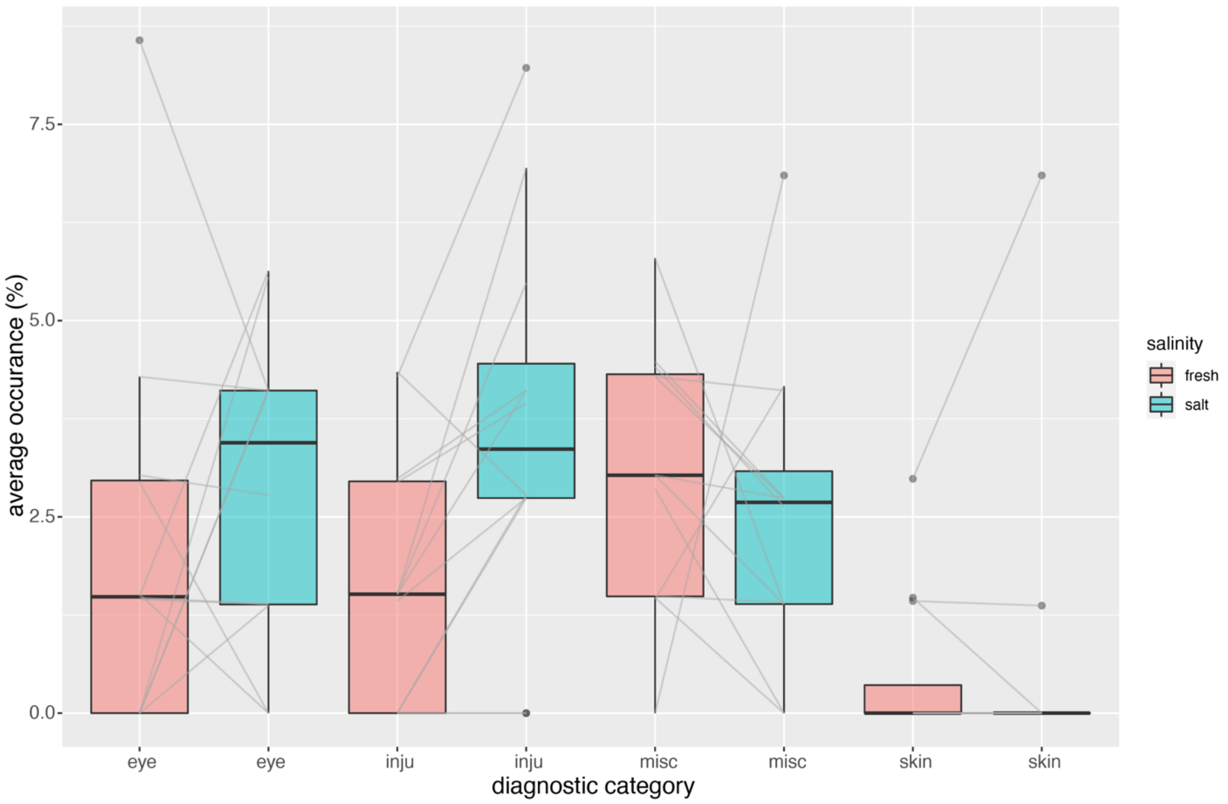

Figure S1. Average occurrence of certain diagnostic findings for the fresh- (red) and saltwater (green) facilities, including. For each month, we calculated the average occurrence of specific diagnoses over a ten-year period, adjusted for the total number of months each individual lived in the facility. Each boxplot displays the variation of the occurrences for the different month of a year which are connected with a gray line in between the fresh- and saltwater category. Note that some lines are double, which is not always visible. X-axis labels: eye = eye disease, inju = injuries, skin = skin disease and misc = other diagnoses.

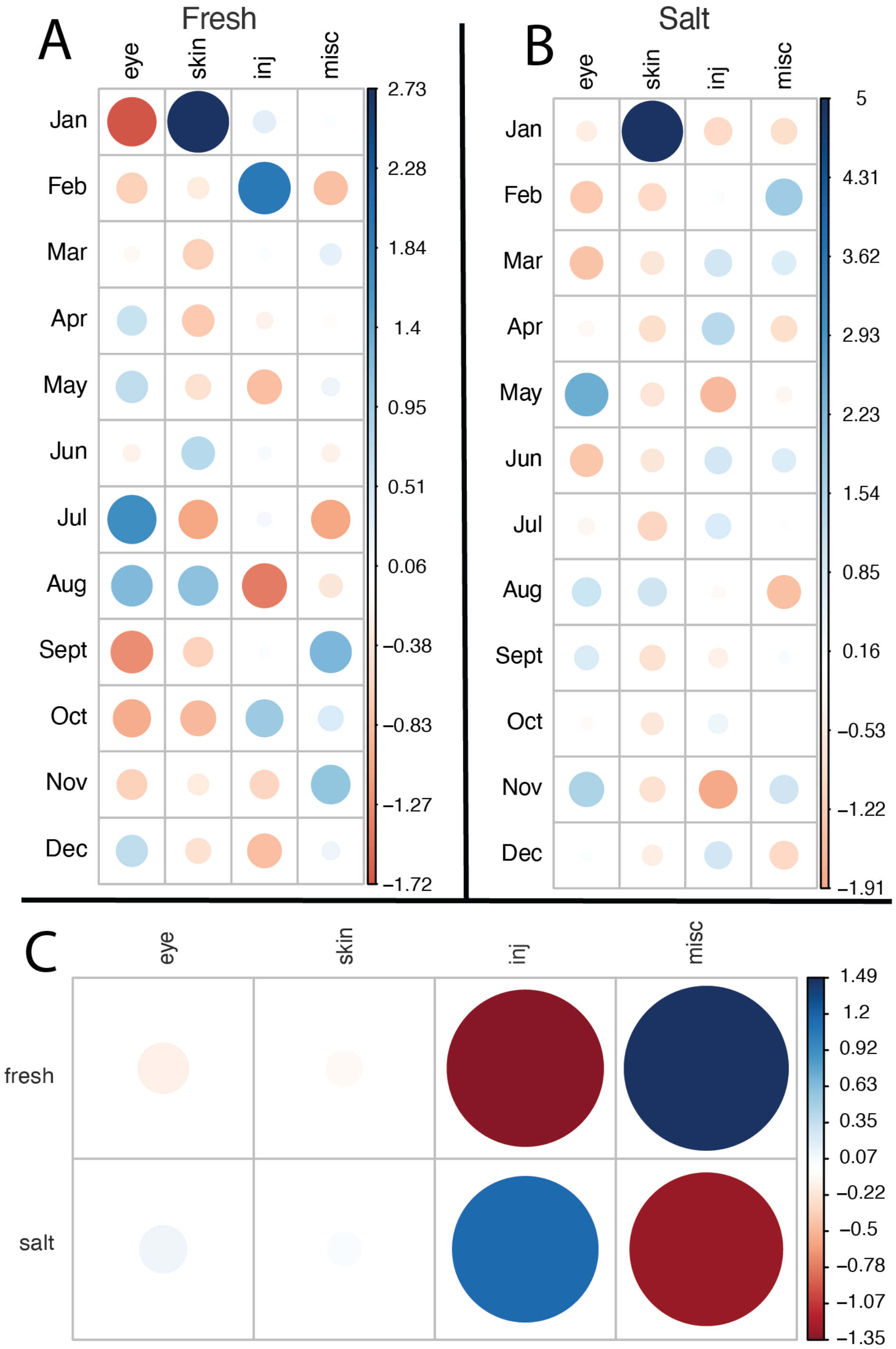

Figure S2. Pearson residuals from the expected values for the average occurrences (percent per month) of certain diagnostic categories. Values are based on the time the animals lived in the respective facility. Positive residuals are indicated in blue and specify an attraction (positive association) between the corresponding row and column variables. Negative residuals are indicated in red and imply a repulsion (negative association) between the corresponding row and column variables. The size of the circle is proportional to the amount of the cell contribution. (A) Residual plot for the fresh water facility. (B) Residual plot for the salt water facility, note that the residuals are very high compared to the other plots. (C) Residual plot directly comparing salt and fresh water facilities.

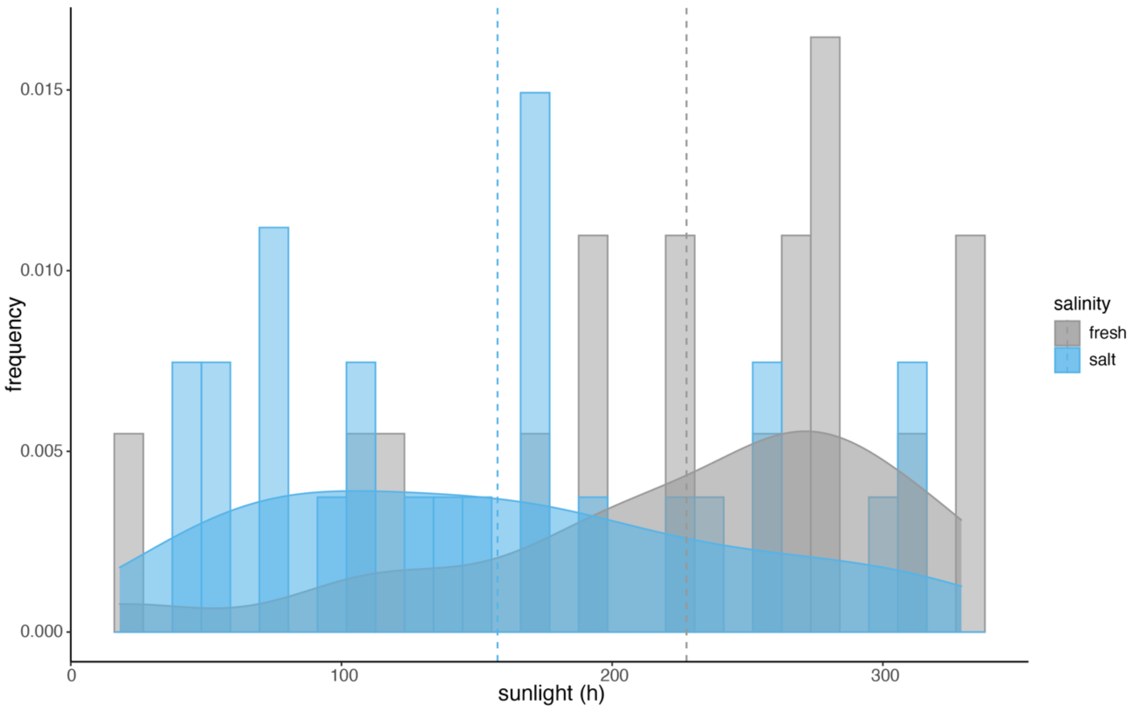

Figure S3. Frequency of ocular diseases during different amounts of hours of sunshine. The saltwater facility is indicated in blue; the freshwater facility is indicated in gray. The dashed lines indicate the mean values. If UV radiation had an influence on the occurrence of ocular diseases, there should be a similar distribution for fresh and saltwater.

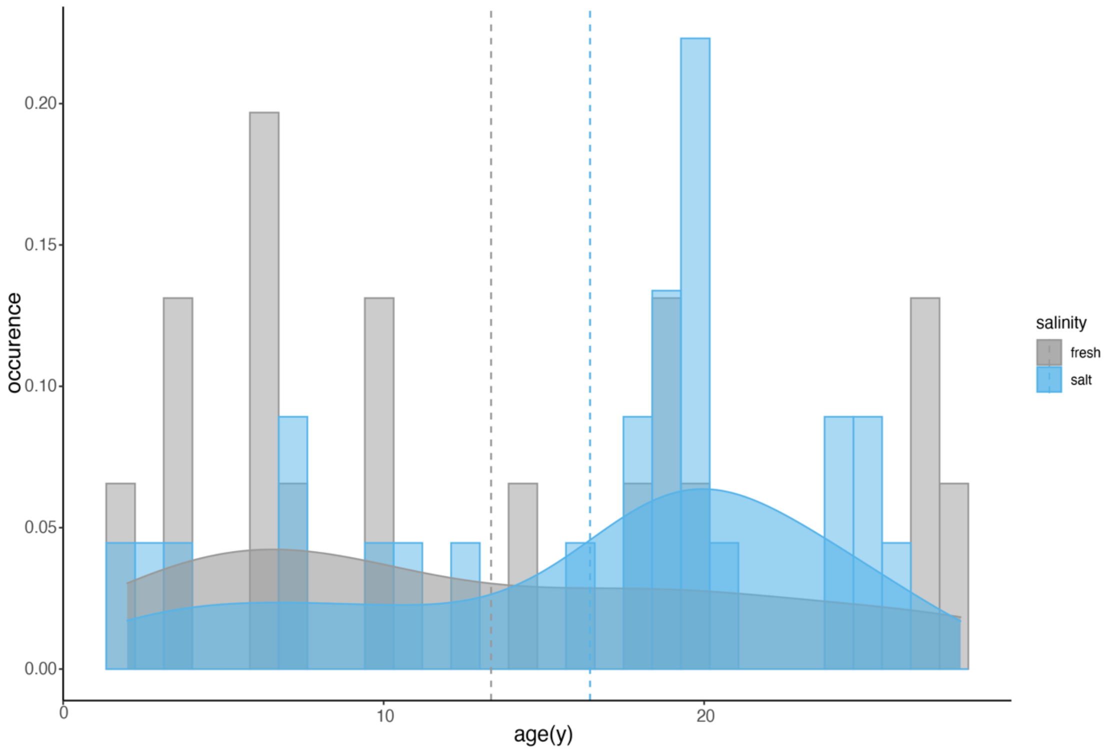

Figure S4. Distribution of age of the California sea lions which were diagnosed with an ocular disease**.** The saltwater facility is indicated in blue; the freshwater facility is indicated in gray. The dashed lines indicate the mean values.
